# The Long-Term Presence of SARS-CoV-2 on Cold-Chain Food Packaging Surfaces Indicates a New COVID-19 Winter Outbreak: A Mini Review

**DOI:** 10.3389/fpubh.2021.650493

**Published:** 2021-05-20

**Authors:** Yuhua Chi, Qingxiu Wang, Guosheng Chen, Shiliang Zheng

**Affiliations:** ^1^General Practice Teaching and Research Section, Weifang Medical University, Weifang, China; ^2^Department of General Practice, Affiliated Hospital of Weifang Medical University, Weifang, China; ^3^Department of Infection Management, Affiliated Hospital of Weifang Medical University, Weifang, China; ^4^College of General Practice, Weifang Medical University, Weifang, China

**Keywords:** COVID-19, SARS-CoV-2, cold chain, item surface, outbreak

## Abstract

Severe acute respiratory syndrome coronavirus type 2 (SARS-CoV-2) is a highly infectious virus that is transmitted primarily through droplets or by coming in close contact with an infected person. In 2020, there was a global outbreak of COVID-19, resulting in an unprecedented global burden of disease, health care costs, and had a significant economic impact. Recently, SARS-CoV-2 was detected on the outer packaging of imported cold chain items in China and has led to virus transmission events, causing great concern. This paper analyses the factors of SARS-CoV-2 survival and transmission in different places and environments, especially the characteristics of low temperatures and object surfaces. It was found that SARS-CoV-2 could survive on surfaces of cold and moist objects in the cold chain for more than 3 weeks, potentially causing COVID-19 transmission. We believe that the low-temperature environment in winter may accelerate the spread of the outbreak and new outbreaks may occur. Overall, SARS-CoV-2 transmission that is susceptible to low winter temperatures is critical for predicting winter pandemics, allowing for the appropriate action to be taken in advance.

## Introduction

Coronavirus Disease 2019 (COVID-19), discovered in December 2019, is a new respiratory infectious disease that has spread faster and in greater numbers than anyone had initially anticipated (1, 2). As of November 25, 2020, the cumulative number of confirmed cases exceeded 60 million and deaths exceeded 1.4 million. The COVID-19 pandemic not only threatens human health but also severely stalled global economic development. China, which was the first country to report the disease, however, brought the epidemic under by April 2020 and implemented the normalization of epidemic prevention and control in May 2020. The focus of normalized outbreak prevention and control in China includes the timely detection, isolation, and treatment of imported cases. Recently, imported cold-chain foods and its packaging surfaces were inspected for the novel SARS-CoV-2, and there have subsequently been incidents of infection with SARS-CoV-2 in related staff members, causing further transmissions (3). This situation is of great concern and is receiving attention from relevant management and people in China, and corresponding countermeasures have been taken (4, 5). At present, there is an increased concern regarding the upcoming winter season and the potential of a new winter outbreak. We conducted a systematic search on 25 November 2020 using PubMed, Web of Science, EMBASE, and CNKI. The search terms included “2019nCoV,” “SARS-CoV-2”, “COVID-19,” “survival rate,” “cold chain,” “article surface,” and “winter,” “outbreak,” and a total of 368 relevant articles were searched. Four cold chain food circulars and policies were searched for in the CDC weekly bulletin and the official website of the State Council. Two authors discussed the extracted information and screened 30 of them for further analysis. The impact of different environmental factors such as low temperature on virus survival and transmission were comprehensively analyzed, and the public were reminded, to strengthen the awareness of the prevention and control of COVID-19 in winter.

### The Impact of Different Sites on the Survival and Transmission of SARS-CoV-2

Hospitals are the most common areas for SARS-CoV-2 survival and transmission, especially in fever clinics, isolation wards, and intensive care units. Infectious disease clinics, respiratory clinics, emergency departments, pediatric clinics, pre-screening triage areas, and imaging departments are also high-risk areas for SARS-CoV-2 survival and transmission in hospitals. Jiang et al. (6) studied isolation wards, outpatient fever clinics, intensive care units, and nurses stations with 28 air samples and 130 surface samples. The RT-PCR test results showed a SARS-CoV-2 positivity rate of 1/28 (3.57%) and at the nurses station it was 1/130 (0.77%). During the COVID-19 pandemic, normal medical procedures at hospitals in many countries were disrupted, some elective surgeries were postponed, and all hospital chronic disease consultations were significantly reduced, which in turn reduced the spread of SARS-CoV-2 to some extent. Unfortunately, during the early stages of the epidemic, nosocomial infections within hospitals were significantly increased due to mutual infections among doctors, nurses, and patients. Since then, with increased awareness of protection and isolation and protection facilities in place, nosocomial infections have declined significantly.

Frequent contact with contaminated surfaces in public places is a potential route for SARS-CoV-2 transmission. Crowded public places during the epidemic are also high-risk areas for the presence and spread of the virus, as these areas may include people with latent SARS-CoV-2 and asymptomatic infections, or even people with SARS-CoV-2. Unlike other respiratory infections, the risk of spreading the disease in latent patients is the same as infected individuals who are asymptomatic (7, 8), which may lead to latent patients entering public places under the misconception that they are not contagious and thus cannot infect others. Among these public places, confined and crowded buses, airplane cabins, subways, supermarkets, restaurants, airports and train stations are areas that pose a greater risk to people becoming infected. A report showed that one COVID-19 patient and 68 healthy individuals took the same bus; 24 (35.3%) of those healthy individuals were later diagnosed COVID-19 (9). Airports pose an even greater risk than buses, and are not only densely populated and mobile, but are likely to have passengers and people who are asymptomatic or latent coming from areas with higher confirmed cases of SARS-CoV-2 infection, enabling the acceleration of widespread transmission to large numbers of mobile people. Multiple locations in airports such as escalator handrails made of plastic, elevator buttons, and security trays associated with high exposure rates are likely to have surface contamination (10). Air transport can accelerate and amplify the spread of viruses in aircraft because the air in the confined cabin, on the handrails of aircraft seats and their tables, is likely to contain SARS-CoV-2 that can easily lead to intensive contact and air aerosol transmission of infections in dense spaces, which may be more common and more important than airport transmission.

### The Impact of Different Environments on the Survival and Spread of SARS-CoV-2

The virus relies on the host's cellular machinery to produce RNA and build proteins for its own use to replicate and grow. The virus cannot reproduce outside the host and can only survive for a short time away from body fluids, during which time infection is possible.

The survival time of viruses outside of the body is strongly influenced by environmental factors. In addition to the surface of an object, the survival time is mainly related to climate, light, temperature, and humidity. The virus is also sensitive to dryness, sunlight, ultraviolet light, and even air quality (11–13). The survival time of viruses on the surface of substances varies with different temperatures, different air, and with different humidity. The higher the temperature, the more difficult it is for the virus to survive, with 56°C for 30 min inactivating all viruses. At room temperature, around 25°C, viruses quickly lose their infectivity, but in winter, they survive longer *in vitro* (14, 15). Viruses are usually more likely to spread in dry air, but this does not mean that they survive longer in dry air. This is because in moist air, viruses are more likely to land on the surface of an object you touch and on the ground, but are more likely to float in dry air. It is therefore not contradictory, that it is more likely to survive in moist air. Studies have found no reduction in SARS-CoV-2 titers or its ability to survive for 3 weeks in meat of inoculated chicken, pork, and salmon filets stored at −20°C (16).

Although, temperature has a greater effect on virus survival than relative humidity, humidity also plays a role. SARS-CoV-2 survives longer at room temperature with a 50% relative humidity than in 30% relative humidity (17). A study confirmed that SARS-CoV-2 survived in Dulbecco's modified medium (DMEM) at 4°C, 22°C, and 37°C for 14, 7, and 1 day, respectively (18).

### The Influence of Material Surface Characteristics on the Survival and Spread of SARS-CoV-2

The persistence of respiratory viruses in the external host environment is key to allowing their transmission, and the characteristics of virus transmission in addition to its own properties, depend primarily on the physicochemical properties of the surface of the material it contaminates and the environmental conditions it is exposed to, which are also important factors in determining the extent of virus transmission (19). These are “soft surface” materials such as, cardboard, paper, and fabrics, where the survival time of the virus is relatively short; “Hard surface” materials, such as, plastics and steel, have longer virus survival times (20).

A recent study by Chin et al. (18) reported that SARS-CoV-2 survived at room temperature with 65% relative humidity for 7 days on plastic surfaces and 7 days on stainless steel surfaces before complete inactivation, was infectious on glass for 2 days, and was completely undetectable after 4 days. During the outbreak, SARS-Cov-2 was detected in garbage cans, on the floor, on computer mice used by doctors and nurses, in corridors, and on handrails of hospital beds in high-risk areas of the hospital (6).

SARS-CoV-2 exhibits different persistence of survival on different porous surfaces. In general, it survived longer (a few days) on surfaces with high porosity than on those with low porosity. The relative survival times of commonly used word processing paper, paper documents, banknotes, and mail wrappers were relatively short, with viruses found on the inner and outer layers of masks at room temperature with a relative humidity of 65% for 4 and 7 days, respectively, and even on the outer layer of surgical masks after 7 days (18). The survival of SARS-CoV-2 on the surfaces of different objects varied considerably, as shown in Table 1.

**Table 1:** The survival time of SARS-CoV-2 on the surface of different objects.

**References**	**Type of object surface**	**Humidity**	**Temperature ^**°**^C**	**Duration**
Chin et al. (18)	Stainless steel	65%	22	4 d
van Doremalen et al. (20)	Stainless steel	NR	NG	3 d
Chin et al. (18)	Glass	65%	22	2 d
Chin et al. (18)	Wood	65%	22	1 d
van Doremalen et al. (20)	Plastic	NG	NG	3 d
Chin et al. (18)	Plastic	65%	22	4 d
Chin et al. (18)	Cloth	65%	22	1 d
Chin et al. (18)	Surgical mask outer layer	65%	22	7 d
Chin et al. (18)	Surgical mask inner layer	65%	22	4 d
Chin et al. (18)	Banknotes	65%	22	2 d
Chin et al. (18)	Paper	65%	22	30 min
Fisher et al. (16)	Chicken, pork and salmon filets(VL)	NR	−20 (Frozen)	21 d or more
Central People's Government of the People's Republic of China (5)	Cold-chain seafood	NR	−18	21 d or more

### SARS-CoV-2 Survives on the Packaging Surfaces of Cold-Chain Products for Long Periods of Time

Different countries and regions implement their own food safety management systems during the production and packaging of cold-chain products, for example, hygienic practices such as wearing clean clothing, washing hands before handling, and wearing head coverings and masks to prevent any potential microbial contamination to the food (21). It has been reported that ~90% SARS-CoV-2 transmission is due to symptomatic and asymptomatic patients, with the remaining 10% coming from the environment, including contaminated surfaces (22). Thus, person-to-person transmission of the virus in the food environment, including manufacturing, retail, and food service industries, remains the greatest risk. This emphasizes the importance of wearing appropriate personal protective equipment and practicing proper hand hygiene and social distancing.

Chin et al. (18) also found a similar resistance of the virus to refrigeration, with little reduction in infectivity of SARS-CoV-2 when the transport material was kept at 4°C for 14 days, which was not previously expected. Therefore, it is likely that the virus survived longer at refrigerated temperatures in some substances. Despite the protective effect of the−18°C cold chain environment on food, it is likely to lead to the prolonged survival of SARS-CoV-2 under such conditions.

### SARS-CoV-2 Transmission Through the Cold-Chain and Its Outer Packaging Surface

Between 22 and 23 July 2020, three local COVID-19 cases were reported in Dalian City, Liaoning Province, China. All three patients reported that they did not leave Dalian 14 days before the onset of disease and had no COVID-19 case contact history and no foreign personnel contact history. Epidemiological investigation, medical isolation, and nucleic acid detection was immediately carried out in Dalian, and 12 asymptomatic infections were detected from close contacts with Patient 1. Because asymptomatic infections make up a large proportion of total infections, the outbreak was likely observed at its beginning stages. Most newly confirmed cases were those that had been diagnosed as having asymptomatic infections but had onset of symptoms during quarantine. The Dalian outbreak was likely related to the processing of cold chain seafood products, in particular imported contaminated products. Therefore, the surveillance of imported COVID-19 should be strengthened, especially for the detection and monitoring of nucleic acids of the COVID-19 virus in imported foods, and a scientific and routine mechanism for entry detection should be implemented (23).

In Qingdao, China, two imported food processors has positive SARS-CoV-2 nucleic acid tests and were later diagnosed with COVID-19 when they presented symptoms. Simultaneously, SARS-CoV-2 was detected in the imported food and on the surfaces that were handled by the two workers. On 17 October 2020, The China Center for Disease Control and Prevention officially confirmed the first extra-laboratory SARS-CoV-2 infection transported in the cold-chain under special conditions (3). The virus can survive for a longer period of time on the outer packaging of these items, suggesting that SARS-CoV-2 has the potential to be imported across borders over long distances, using cold-chain items as carriers.

### SARS-CoV-2 Transmission Through the Cold-Chain and Its Outer Packaging Surfaces Suggests the Possibility of New Outbreaks in Winter

At the start of the COVID-19 pandemic, it was too early to think that SARS-CoV-2 would become a seasonal virus. However, there is growing evidence that small seasonal effects may lead to larger outbreaks in the winter, based on knowledge of how the virus spreads and how people behave in the colder months (24, 25). During the winter months, much of the world is exposed to cold air and low temperatures, which provide suitable conditions for the survival and propagation of SARS-CoV-2 (26); The winter world acts as a “large cold-chain environmental reservoir” where SARS-CoV-2 can easily survive and spread. Combined with extreme winter weather and climatic conditions, frequent interactions between people indoors in poorly ventilated areas during the colder months could easily cause the spread of SARS-CoV-2, leading to outbreaks of COVID-19 (27).

The main advantage of this paper is that we analyzed the impacts of different sites, different environments, and material surface characteristics on the survival and transmission of SARS-CoV-2, and that SARS-CoV-2 can be transmitted through the cold-chain and its outer packaging surfaces. But transmission through the cold-chain and its outer packaging surfaces is limited to a small number of cases, and systematic clinical studies and laboratory information remains lacking. Even so, it is sufficient to suggest that SARS-CoV-2 survives for a long time in a cold environment, leading to the transmission of COVID-19. Therefore, the management of imported cold-chain foods and its practitioners should be strengthened, and cold-chain food management policies and specific measures should be strictly implemented to promptly detect and isolate those who come into contact with SARS-COV-2 detected in the outer packaging of imported cold-chain foods to avoid the spread of COVID-19. We can prepare for the arrival of winter by developing and strictly enforcing preventive measures and health policies, by going out less, social distancing, wearing masks when going to public places, and maintaining hand hygiene, so that we may avoid new outbreaks of the SARS-CoV-2 epidemic in winter (28–30).

In conclusion, the fact that SARS-CoV-2 can be transmitted through the cold-chain and its outer packaging surfaces further reveals that cold temperatures in winter might lead to the spread of the disease, which is crucial for predicting a winter pandemic and to take the appropriate measures to control its spread in advance.

## Author Contributions

YC was responsible for organizing the literature. QW for data analysis. GC for organizing the tables. SZ for providing the overall idea. All authors contributed to the article and approved the submitted version.

## Conflict of Interest

The authors declare that the research was conducted in the absence of any commercial or financial relationships that could be construed as a potential conflict of interest.
